# Relationship of Physical Activity and Sedentary Time with Metabolic Health in Children and Adolescents Measured by Accelerometer: A Narrative Review

**DOI:** 10.3390/healthcare9060709

**Published:** 2021-06-10

**Authors:** Jungjun Lim, Joon-Sik Kim, Soyoung Park, On Lee, Wi-Young So

**Affiliations:** 1 Department of Physical Education, College of Education, Seoul National University, Seoul 08826, Korea; imjung87@naver.com (J.L.); joonsik@snu.ac.kr (J.-S.K.); thdud0623@snu.ac.kr (S.P.); 2 Korea Institute of Sports Science, Seoul 01794, Korea; 3 College of Humanities and Arts, Korea National University of Transportation, Chungju-si 27469, Korea

**Keywords:** accelerometer, adolescents, metabolic health, physical activity, sedentary time

## Abstract

The purpose of this study was to summarize the associations of physical activity (PA) and sedentary time (SED) with metabolic health and examine the effects of time reallocation on metabolic health in adolescents using accelerometer data. A literature search was conducted using PubMed, ScienceDirect, Web of Science, Cochran Library, and Google Scholar, and 27 articles were reviewed. Recent research generally confirms the associations of PA and SED with metabolic health. High PA levels and low SED levels had a positive relationship with metabolic health. Moreover, reallocating 10 min of daily SED to PA was associated with better metabolic health indicators. These results were stronger for moderate-to-vigorous physical activity than for light intensity PA. Thus, efforts to convert SED into PA of at least moderate intensity appear to be an effective strategy to prevent metabolic disease development in children and adolescents. However, some of the associations between PA and metabolic health indicators were inconsistent, depending on age, obesity degree, and PA intensity. Additionally, various accelerometer data collection and processing criteria impact the interpretation of the results. Therefore, consistent accelerometer data collection and analysis methods are needed in future studies. Further, intervention studies are required to verify the causality and effectiveness of the isotemporal substitution model.

## 1. Introduction

The World Health Organization (WHO) guidelines on physical activity (PA) and sedentary behavior for children and adolescents (aged 5–17 years) recommend that individuals should engage in at least an average of 60 min/day of moderate-to-vigorous physical activity (MVPA), mostly aerobic. In addition, children and adolescents should restrict the amount of time spent being sedentary [1]. The Physical Activity Guidelines Advisory Committee (PAGAC) scientific report suggests 500 to 1000 metabolic equivalent (MET)-minutes of MVPA/week (or 150 to 300 min/week) [2]. Studies suggest that approximately 24% of children (aged 6–17 years) participate in 60 min of PA every day [3], and 33% engage in 2 h or less of screen time per day [4]. These statistics indicate that a large percentage of children and adolescents do not follow the guidelines set forth by the WHO and PAGAC.

The associations of PA and sedentary time (SED) with health indicators in children and adolescents have been well-established. A systematic review of PA and SED indicated that children and adolescents who engaged in high levels of PA and low levels of SED had better adiposity and cardiometabolic health than those with low PA and high SED [5]. In adult obesity, only the size (hypertrophy) of fat cells increases. However, obesity in childhood and adolescence can increase the size and the number (hyperplasia) of fat cells. Therefore, obesity in childhood and adolescence is likely linked to adult obesity. Some studies indicate that adults are more than 80% likely to have obesity if they had obesity in childhood or adolescence [6,7], and obesity is a major cause of increased incidence of mortality from non-inflammatory diseases, such as high blood pressure, diabetes, hyperlipidemia, and cardiovascular disease [8,9,10]. Adolescence, which involves multiple rapid changes, is a crucial time for determining important health behaviors, as the lifestyle acquired during this period is difficult to change [11].

Researchers use various methods to measure PA, including self-reported questionnaires, pedometers, heart rate monitors, and accelerometers. The tool should be selected considering the goal, feasibility, and validity of the study [12]. Among the various tools, the questionnaire, which is a subjective measurement tool, is widely used in large-scale cohort studies at the national and community levels because it is cost effective and easy to administer. However, the use of a questionnaire involves potential problems, such as distorted estimation of PA and SED because of recall bias and social desirability [13,14,15]. An accelerometer is an objective measurement device that converts the magnitude of acceleration generated by body movement into an electrical signal through an embedded piezoelectric device, which can classify the intensity of PA according to the size of the signal [16]. The amount of daily SED and PA is calculated by classifying activity counts over a specific time interval (epoch) into a series of cut-off points. Therefore, the accelerometer, which measures all movements of the subject, is more accurate than questionnaires and compensates for problems such as recall bias and social desirability [13,15]. The use of accelerometers in studies is increasing, as they are reasonably priced [17] and a feasible alternative to questionnaires, especially in children and adolescents [18]. It is, therefore, being used in the continuous efforts to accurately determine the level of youth PA.

The National Health and Nutrition Examination Survey (NHANES) and the Canadian Health Measures Survey (CHMS) examined the PA of adolescents using accelerometers. In both studies, large differences were found between PA time measured through questionnaires and accelerometers [19]. Moreover, PA had stronger associations with cardiovascular disease and metabolic syndrome when it was measured using accelerometers than questionnaires [20,21].

As time is limited and finite, spending it on one movement-related behavior could change other behaviors. The isotemporal substitution model (ISM) is increasingly used to identify the association of PA and SED with various health variables. The ISM has been proposed as a mathematical technique to model the impact of reallocating time spent in one behavior (e.g., SED) with another behavior (e.g., MVPA) for an equal amount of time [22]. This theoretical method allows a more realistic approach to understanding the effects of replacing SED with various intensities of PA [23].

The purpose of this literature review is to (1) summarize the association of PA and SED with metabolic health and (2) examine the statistical relationships with reallocating time between categories of different PA intensities and metabolic health in children and adolescents among accelerometer studies.

## 2. Methods

We adopted the Population, Exposure, Comparison, Outcome (PECO) approach. The population comprised children and adolescents. The exposure was people who were physically active, the comparison was people who were inactive or had high sedentary behavior, and the outcome was metabolic health variables. To identify the associations of PA and SED with metabolic health in children and adolescents, the following electronic databases were searched through February 2021: PubMed, ScienceDirect, Web of Science, Cochran Library, and Google Scholar. We searched for terms that reflected exposure variables of interest (e.g., PA, sedentary behavior, SED, accelerometer-measured, objectively measured), cardiometabolic-related variables (e.g., health, cardiometabolic, metabolic, obesity, and biomarkers), and methods (e.g., isotemporal substitution, replacing time, reallocating, ANOVA, correlation, and multiple regression analysis).

Articles were selected for detailed analysis based on the following inclusion criteria: (1) school-aged children or adolescents under the age of 19 years with no known physical health restrictions; (2) accelerometer-measured volume and/or pattern of sedentary behaviors/PA and its association with one or more cardiometabolic outcomes, including body mass index (BMI), waist circumference, percentage body fat (%BF), blood pressure (BP), and blood-related biomarkers; (3) published, in the press, or accepted original articles in the English language, between January 2000 and February 2021. RCT studies that applied separate interventions were excluded. Additionally, review articles, meta-analyses, and systematic reviews summarizing conclusions of PA/sedentary behavior and their associations with cardiometabolic indicators were excluded due to difficulties in determining the isolated effect of PA/sedentary behavior.

Titles and abstracts of identified publications were screened by three authors (J.L., J.-S.K., S.P.). If abstracts provided insufficient data or were not available, the entire paper was retrieved and then screened to determine whether it met the inclusion criteria. Disagreements were resolved through discussion among the three authors (J.L., J.-S.K., S.P.). The following information was extracted from the included articles: authors, study design, country, sample size, age, outcomes, and result. Data extraction was performed independently by each of the three authors (J.L., J.-S.K., S.P.). At each part, extracted data were cross-checked by the other two authors (O.L., W.-Y.S.). Studies targeting children and adolescents were selected. In cases where other age groups, such as adults or preschoolers were included in the same study, the data were scrutinized and analyzed appropriately.

## 3. Results

**Figure 1:**
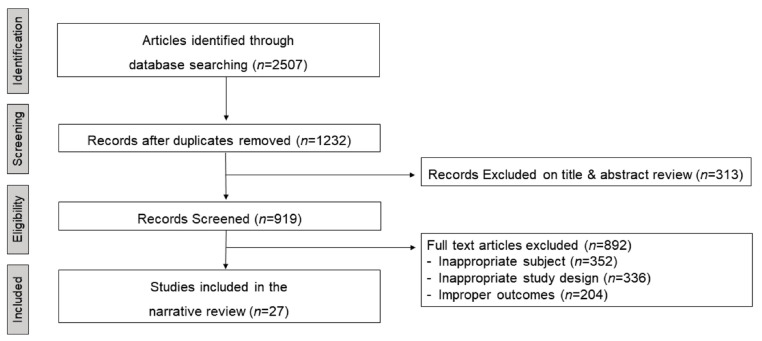
Flow chart of the identification and selection of studies for inclusion.

The results of the literature search are presented in Figure 1. The initial database search yielded 2507 results, of which 1275 were excluded owing to duplicate records and 313 on the basis of title and abstract review. An additional 892 articles were excluded after screening through the full text, as they did not fulfill the inclusion criteria. The cut-off point for PA intensity was different for each study. First, most studies defined SED as less than 100 counts per minute (CPM), while others defined it as less than 152 and 199 CPM. Cut-off points for light-intensity PA (LPA), moderate-intensity PA (MPA), vigorous PA (VPA), and very vigorous PA (VVPA) used in this review were defined using the prediction equations used in the studies of Migueles et al. [18], Evenson et al. [24], Pate et al. [25], Freedson et al. [26], and Troiano et al. [27]. In addition, both METs and CPM values presented in each study were used. Bouts were defined as 1 min, 1–4 min, 5–9 min, and 10 or more min above the threshold. Therefore, 27 articles were finally included in this review [5,28,29,30,31,32,33,34,35,36,37,38,39,40,41,42,43,44,45,46,47,48,49,50,51,52,53]. Table 1 displays the characteristics of individual studies and study results. The methods used for examining accelerometer data are shown in Table S1. The most frequently used accelerometers in this study were ActiGraph (77%), Actical (15%), activPAL (4%), and Actiwatch (4%). For epoch time, the most frequently used units, in this order, were 60s (50%), 15s (27%), 10s (11%), 1s (8%), and 5s (4%). In most studies (more than 90%), valid data were defined as data for at least 3 days per week and at least 8 h per day. Some studies required a minimum of 1 day of weekend data. More than 60 min of consecutive zero counts were defined as the non-wear time (56%). Some studies (33%) regarded non-wear time as 10 min or more of consecutive zero counts.

### 3.1. Physical Activity, Sedentary Time, and Metabolic Health

Of all the studies investigating the relationship between objectively measured SED and metabolic health indicators in Table 1, 12 studies examined obesity indicators [5,30,31,32,33,34,38,40,43,49,50,51], 5 analyzed BP [30,32,38,49,50], 5 studied blood lipid indicators [5,33,38,49,50], and 8 examined glucose-related indicators [28,29,30,38,41,48,50,53]. In addition, among all the studies that identified the relationship between objectively measured PA and metabolic health indicators, 10 of them examined obesity indicators [28,29,35,38,40,41,47,50,51,53], 9 studied BP [29,30,35,38,41,47,48,50,53], 8 analyzed blood lipid indicators [29,30,35,38,47,48,50,53], and 8 examined glucose-related indicators [28,29,30,38,41,48,50,53].

#### 3.1.1. Markers of Obesity

Numerous cross-sectional and longitudinal studies showed positive associations between the volume of objectively measured SED [5,30,31,32,33,34,38,40,43,49,50,51], PA [28,29,35,38,40,41,47,50,51,53], and obesity variables, including BMI, WC, and %BF.

First, out of 12 cross-sectional and longitudinal studies that investigated the relationship between objectively measured SED and BMI in children and adolescents, 8 studies showed a positive relationship of SED with BMI [5,31,32,34,40,43,50,51]. A large-scale study conducted by Katzmarzyk et al. [40] with 6539 children and adolescents in 12 countries demonstrated that an increase of 70 min in SED increases the incidence of obesity among children and adolescents by 1.19 times. Further, it was consistent with the results by Chinapaw et al. [33]. Interestingly, Carson and Janssen [32] reported that BMI increased with increasing SED only in the lower PA group, and not in the group with higher PA.

In a longitudinal study by Mitchell et al. [43], which confirmed the relationship between SED and BMI in children and adolescents over a period of 6 years, the daily SED of children and adolescents increased with increasing age. In addition, it was reported that this increase in SED significantly increased BMI irrespective of MVPA and other confounding variables [43], supporting the results of a study by Saunders et al. [5] that breaking SED can lower children’s BMI [5]. Contrarily, according to a longitudinal study of 1826 adolescents aged 12–15 years studied for 15–17 years, an increase in SED was associated with a decrease in total cholesterol (TC) and triglycerides (TG) but was not associated with BMI [30].

Second, among the indicators of obesity, four of six cross-sectional studies found a positive relationship between objectively measured SED using accelerometer and WC in children and adolescents [5,33,34,50]. In both Strizich’s study [50] and Saunders’ study [5] with children with a family history of obesity, an increase in SED was associated with poor WC. Colley [34] reported a positive correlation between spending more than 40 min of SED after 3 p.m. and an increase in WC. For SED longer than 60 min, it was reported that the WC increased by 3.4 cm. In Stockwell’s study [49], the number of sedentary breaks showed a significant negative correlation with WC, consistent with previous findings; however, increasing SED was associated with decreased WC, showing contradictory results from previous studies. Ekelund et al. [38] also reported that SED did not show a significant relationship with WC among various metabolic disease-related indicators.

Third, two of the three studies that analyzed the relationship between SED measured by accelerometer and body fat in children and adolescents showed a positive correlation between SED and body fat (e.g., fat mass, %BF) [38,51]. In a cross-sectional study of 130 rural children and adolescents, Treuth et al. [51] found a positive association between SED measured using an accelerometer and %BF measured using bioelectrical impedance analysis only in girls. A similar tendency was observed when measuring body fat using skin fold by Ekelund et al. [38].

In the Stockwell et al. study [49], sedentary breaks were negatively correlated with %BF, supporting the findings of a previous study [38,51]. Conversely, in the case of SED, there was a negative correlation with %BF, contradicting the results of previous studies [38,51].

The 2003–2006 NHANES of 3165 children and adolescents aged 6–18 years attempted to investigate the relationship between the MVPA bout pattern and metabolic syndrome risk factors. Participants were classified into quartiles according to bout duration. PA was analyzed using three cut-off points (Evenson, Freedson/Saint-Maurice, Freedson/Troiano). Regardless of the cut-off point, those with longer continuous bouts of MVPA showed significantly lower BMI, WC percentile, and WHR than those with shorter bouts of MVPA [53]. In a cross-sectional study confirming the associations between PA and cardiometabolic risk factor of 1426 youth aged 8–16 years, PA was classified into tertiles and analyzed. BMI and WC were higher in the 3rd tertile group with the lowest PA [50]. In addition, according to a study by Colley et al. [35], a 1-h increase in MVPA time was statistically related to a decrease of 1.2 kg/m^2^ (−0.020(β) × 60 min) in BMI and a decrease of 3.2 cm (−0.054(β) × 60 min) in WC.

In the Nguyen et al. study, the metabolic syndrome prevalence was found to be 3.6 times higher than normal BMI (3.3%) [47]. Similarly, in a study of students 7–19 years old (e.g., elementary, middle, and high school) in rural areas, overall PA intensities, including LPA, MPA, and VPA, were negatively correlated with BMI, fat mass, and %BF at all ages, excluding only BMI of elementary school students [51]. In a study of 6538 children from 12 countries, a statistically negative relationship of obesity with MVPA and VPA was found in all the countries [40].

#### 3.1.2. Blood Pressure

Some observational studies investigated the associations among the volume of objectively measured SED [30,32,38,49,50], PA [29,30,35,38,41,47,48,50,53], and BP. To date, the BP responses according to the level of SED may show controversial results depending on age, sex, and the measuring instrument.

Some studies suggested that SED has a positive association with an increase in systolic blood pressure (SBP)/diastolic blood pressure (DBP) [38]. In the European youth heart study, an increase in SED was shown to increase both SBP and DBP in children and adolescents [38]. Similarly, Stockwell et al. reported a negative relationship between the number of sedentary breaks and BP. In contrast, SBP presented a negative association with SED, showing a different pattern from previous studies [49].

In Strizich’s cross-sectional study [50], a null association was found between SED and BP among 426 Hispanic and Latin youths. In addition, when Carson and Janssen [32] measured SED using subjective and objective methods, there was no significant association with BP in both SED and the number of sedentary breaks [32]. Similarly, a longitudinal study of 1826 adolescents conducted over 15–17 years reported that SED was not associated with SBP/DBP [30].

A study conducted to confirm the association between PA and metabolic syndrome in adolescents reported that the BP of overweight adolescents was significantly higher than that of normal-weight adolescents [47]. Similarly, in the Colley et al. study on children aged 6–11 years, SBP decreased significantly as MVPA increased [35]. In a cross-sectional study conducted with 1709 European youth, PA was found to have significant negative correlations with SBP and DBP in total PA, LPA, MPA, and VPA, regardless of PA intensity [38].

Conversely, some studies showed that the correlation between PA and BP was not significant. In the Healthy Lifestyle in Europe by Nutrition in Adolescence (HELENA) study of 769 adolescents aged 12.5–17.5 years [48] and the cross-sectional studies of Hispanic and Latin children and adolescents [50], PA did not show any significant relationship with either SBP or DBP. In addition, in the Avon Longitudinal Study of Parents and Children (ALSPAC), conducted with 1826 adolescents, PA was not significantly related to SBP or DBP regardless of its intensity [30]. When PA was analyzed using three different MVPA cut-off points, it had no significant correlation with DBP at any of the cut-off points [53].

#### 3.1.3. Blood Lipids

Some cross-sectional, and two longitudinal studies investigated the associations between the volume of objectively measured SED [5,33,38,49,50], PA [29,30,35,38,44,47,48,50,53], and blood lipid profile.

Strizich et al. [50] showed that youth SED had a significant positive relationship with TG, a negative association with high-density lipoprotein cholesterol (HDL-C), and was not significantly correlated with low-density lipoprotein cholesterol (LDL-C). Similarly, in a series of cross-sectional studies, Ekelund et al. [38] reported that SED was not significantly correlated with HDL-C, but had a significant correlation with triacylglycerol, confirming the correlation with some blood lipid markers.

However, some studies showed contradictory results. In a cross-sectional study of 142 Canadian children, SED was not statistically significant in all blood lipid markers, such as LDL-C, HDL-C, and TC [33]. Similarly, in Saunders’ study that targeted obese children with a family history, SED was not statistically significant with HDL-C and LDL-C [5]. In addition, Stockwell et al. [49] reported a significant positive association between the number of sedentary breaks and blood lipid indicators. However, SED had a positive association with HDL-C, contradicting the results of some previous studies.

Kuzik et al. [41] analyzed 4581 participants aged 5–18 years from 6 studies of the International Children’s Accelerometry Database (ICAD) to confirm the correlations among PA, SED, and metabolic health (MU, metabolic unhealthy; MH, metabolic healthy) according to weight status (NW, normal weight; overweight; and obese), and defined metabolic health risk factors as 2 levels (lenient, strict). The results showed that when the MVPA time increased by 10 min, the metabolic health odds ratio significantly decreased by 5% in the NW-MU group and by 6% in the overweight-MU group compared to the NW-MH group as the reference. In addition, calculating the odds ratio by multinomial logistic regression analysis using the MH-NW group as the reference showed that when the MVPA time increased by 10 min, the odds ratio of metabolic health significantly decreased by 8% in the MU-overweight group and by 11% in the MU-obesity group.

Strizich et al. [50] reported that higher levels of MVPA are related to better HDL-C and TG, and the higher the levels of MVPA, the more the TG decreased significantly. Bell et al. [30] reported that higher total PA (CPM) had a strong correlation with HDL-C, but a weak correlation with LDL-C. MVPA also had strong negative correlations with HDL-C and TG, and Saunders et al. reported that PA had no significant effect on HDL-C, LDL-C, and TG [53].

Many studies have confirmed the relationship between PA and blood lipids. However, rather than directly analyzing the relations between PA and indicators of blood lipid, most studies confirmed the relationship between PA and cardiometabolic risk score derived from the calculated value of HDL-C, insulin, glucose, TG, BMI, WC, and BP.

#### 3.1.4. Glucose and Insulin

Some cross-sectional and longitudinal studies have been conducted between SED [5,33,38,49,50], PA [28,29,30,38,41,48,50,53], and diabetes-related markers, including glucose, insulin, and homeostasis model assessment of insulin resistance (HOMA-IR). In a cross-sectional study with 1921 European children and adolescents, a positive association was reported between SED and fasting glucose levels, and SED and insulin levels [38]. In addition, Strizich et al. [50] reported that increased SED is positively associated with insulin and HOMA-IR. Conversely, Saunders et al. [5] found no association between SED and blood glucose in children and adolescents with a family history of obesity. Similarly, in a study by Chinapaw et al. [33], plasma glucose was not significantly associated with SED, which is consistent with the Stockwell et al. findings [49].

Previous studies defined metabolic syndrome according to the International Diabetes Federation (IDF) guidelines. After adjusting for confounding variables, the odds ratio was 5.3 times higher in the lowest MVPA group (4 quartiles; <43 min/day) than in the highest MVPA group (1 quartile; >103 min/day). Compared to the first quartile (MVPA > 103min/day), the odds ratio of the second quartile (64–103 min/day) was significantly higher by 1.1 times and that of the third quartile (43–63 min/day) was significantly higher by 3.9 times [47]. A cross-sectional study confirmed the association between the WC to height ratio and homeostasis model assessment (HOMA) in 11,853 people aged 5.8–18.4 years; the association gradually strengthened as the level of MVPA increased up to 4000–5000 CPM [28]. Higher levels of MVPA had a positive effect on fasting insulin and HOMA-IR, and the higher the level of MVPA, the more fasting insulin and HOMA-IR significantly decreased throughout the tertile [50].

### 3.2. Effects of Replacing Sedentary Time with Physical Activity on Metabolic Health

Recent research has strengthened the evidence for the association between ISM and metabolic health, as seen in eight cross-sectional studies included in Table 1. Among these studies, seven examined obesity indicators [36,37,39,42,44,45,46], three studied BP [39,44,45], and five examined blood lipid indicators and glucose-related indicators [39,44,45,46,52].

#### 3.2.1. Markers of Obesity

Moura et al. [45] objectively measured Brazilian male adolescents’ daily behavioral patterns for 7 days using ActiGraph. ISM was used to evaluate the effects of time reallocating various sedentary bouts (5, 10, 30, and 60 min) with LPA or MVPA on BMI, WC, and %BF. Replacing sedentary behavior with LPA had no relationship with any obesity marker. However, when sedentary behavior was replaced with MVPA, %BF decreased. The effect increased as the replacement time of MVPA increased. BMI and WC had no association with either type of PA.

Meanwhile, the ICAD study, which contained accelerometer-determined PA and SED in children and adolescents from 10 different countries, showed different results [39]. In this study, individuals were classified into adolescents (10–15 years old) and older adolescents (>15 years old) due to the wide age range of the sample and variability in cardiometabolic risk factors by age. When 10 min of daily SED was replaced by 10 min of daily MVPA, WC reduced across all age groups. However, LPA was not significant in older adolescents. Therefore, to reduce cardiometabolic risk factors, engaging in at least MPA appears to be an efficient approach.

In another study, Dalene et al. [36] assessed the time reallocation effects on BMI and WC in adolescents using ISM. As a result, positive effects were observed in BMI and WC when MPA and VPA replaced the 10-min daily SED in 9-year-olds. However, when LPA was replaced by SED, a negative effect was observed. For 15-year-olds, only VPA had a positive effect on BMI and WC, while LPA had a negative effect. Therefore, although the ISM effects may vary depending on age, VPA is shown to be the most effective.

New Zealand’s 2008–2009 National Survey of Children and Young People’s Physical Activity and Dietary Behavior conducted ISM analysis on BMI and found a decrease in BMI when LPA and MVPA replaced 10 min of SED [37]. MVPA had the strongest association with BMI.

Loprinzi et al. [42] examined the association between PA and SED with adiposity markers among children and adolescents using 2003–2006 NHANES data. When 1 h of SED was replaced with LPA or MVPA, adiposity markers such as BMI, WC, and %BF showed positive results only for MVPA in children. There were no significant associations for adolescents.

#### 3.2.2. Blood Pressure

In the Moura et al. study [45], replacing 5–60 min SED with LPA showed positive effects on SBP. However, this result was not significant for any bouts of MVPA. In another study, Hansen et al. [39] reported that when 10 min of SED was replaced with 10 min of daily LPA or MVPA, positive effects were observed in SBP in 10–14-year-olds. However, these results were not observed in 15–19-year-olds. Meanwhile, Moore et al. [44] found that replacing LPA with VPA is negatively associated with SBP and DBP.

#### 3.2.3. Blood Lipids

Verswijveren et al. [52] examined the effects of substituting 10 min of SED with 10 min of total PA (regardless of the manner of accumulation) or 1-min bouts of different intensities of PA on blood lipids such as HDL-C, LDL-C, TG, and TC. Children were categorized into a healthy weight group and an overweight/obese group. After adjusting various covariates, substituting 10 min of sedentary behavior with VPA had positive effects on TG regardless of the degree of obesity. In addition, when sedentary behavior was replaced with VPA, it positively affected HDL-C and TG in healthy-weight children. When sedentary behavior was replaced with MPA, it resulted in better HOMA-IR and HDL-C in healthy-weight and overweight children. Replacing sedentary behavior with VPA, specifically accumulated in 1-min bouts, had no positive effect on HOMA-IR in healthy-weight children.

In contrast, Hansen et al. [39] showed that substituting 10 min of daily sedentary behavior with MVPA had no association with HDL-C. Replacing 10 min of sedentary behavior with LPA showed a static relationship with LDL-C for adolescents aged 10–15 years. However, when MVPA was replaced, it showed a negative relationship. These results disappeared in older adolescents (15–18 years). In addition, TG showed a negative association with MVPA in adolescents and with LPA in older adolescents. Unlike the above results, Moura et al. [45] reported that HDL-C increased only when SED was replaced with LPA, and TG decreased.

In addition, another study by Moura et al. [46] substituted various sitting durations (15, 30, 60, and 120 min) with standing duration on blood lipids. Replacing SED across 15–120 min with standing resulted in statistically significant reductions in serum levels of TC, non-HDL-C, and LDL-C. In contrast, TG increased, showing the opposite effect. There was no significant association with HDL-C.

## 4. Conclusions

Most previous studies found a negative relationship between PA and obesity-related variables. Furthermore, the association was significant even when the intensity of PA was light. In addition, despite the contradictory results of some aforementioned studies, evidence that sedentary behavior indicators (e.g., SED, number of breaks) are positively related to obesity indicators has strengthened over time. Therefore, these findings suggest that reducing SED should be developed as a strategy for managing obesity early in life.

In some studies related to SED and PA, the controversial findings regarding the association with BP may provide limited evidence in this field. Nevertheless, improving PA and reducing SED is essential as it has the potential to reduce the risk of hypertension and related metabolic diseases.

Overall, many studies show significant correlations of PA with HDL-C and TG. Unlike the results of studies examining PA, the association between SED measured using accelerometer and blood lipid variables in this review tends to differ in each study. Therefore, the relationship between the two variables is contested so far, and the possibility that there is no relationship between them cannot be ruled out. However, several previous studies reported associations between SED and blood lipid variables.

There are not many studies analyzing PA and insulin-related variables (e.g., plasma glucose, insulin, and HOMA-IR) separately; however, several studies analyzed PA and insulin-related variables by converting them into a composite index, such as the metabolic risk factor score. These studies reported that MVPA or VPA was significantly correlated with insulin-related indicators. On the contrary, it is suggested that there is no significant correlation between SED and fasting glucose, instead indicating a positive correlation between SED and insulin.

To sum up, our review demonstrates evidence for associations between objectively measured PA and SED with cardiometabolic related variables in children and adolescents. PA was found to have a positive effect on all obesity-related indicators (e.g., BMI, WC, and %BF), HDL-C, and TG, whereas there was a null association with BP, glucose, and insulin. Similarly, a reduction in SED and an increase in sedentary break were found to help reduce obesity and have a positive effect on markers of obesity. Additionally, increased SED has negative effects on insulin in children and adolescents. However, the relationship between BP, blood markers, plasma glucose, and SED was found to be different for each study.

Although the effect was relatively small for some indicators of obesity, notably, the effect of replacing SED with PA using the ISM method on metabolic health was positive for indicators of obesity, BP, blood lipids, glucose, and insulin. These results were stronger for MVPA than for LPA. However, some of the effects on metabolic health were inconsistent, depending on age, degree of obesity, and PA intensity. For example, BMI and WC showed a positive effect when 10-min daily SED was replaced with MPA or VPA in 9-year-olds. Conversely, when LPA was replaced by SED, a negative effect was observed. For 15-year-olds, only VPA positively affected BMI and WC, while LPA negatively affected them. Although TG showed a negative association with MVPA in adolescents, LPA was positively associated with TG in older adolescents. These results are similar to those observed for glucose and insulin, HOMA2-S, and HOMA2-IR. These inconsistent results may have been derived because of the diverse accelerometer raw data collection protocols and data processing criteria.

In summary, increased PA and decreased SED were positively associated with the metabolic health of children and adolescents. Additionally, replacing SED with LPA or MVPA improved the metabolic health of children and adolescents. Therefore, efforts to convert SED into PA, with engagement in at least MPA, appears to be an efficient approach to prevent the development of metabolic disease in children and adolescents.

Several limitations should be considered when interpreting our review. First, the studies included in this review identify a gap in the literature regarding accelerometer-data processing methods. Therefore, careful analysis is needed because different methodological strategies may have interfered with the comparability among studies. Second, in studies using ISM, results are insufficient for some variables, so caution must be exercised when interpreting the results. Third, most of the studies in this review were cross-sectional, so it is difficult to grasp the causal relationship accurately.

Therefore, consistent accelerometer data collection and analysis methods are needed in future studies. Intervention studies are also required to verify the causality and effectiveness of the ISM.

## Figures and Tables

**Table 1:** Associations between PA and SED with metabolic health risk factors in children and adolescents.

Reference	Study Design	Country, n (Sample Size), Age	Outcome	Result
Aadland et al. [28]	CS	Brazil/Denmark/Estonia/Norway/Portugal/Switzerland/UK/US, 11,853, 6–18 Y	BMI, WC, SBP, TC, TG, HDL-C, glucose, insulin, HOMA	Associations with the composite metabolic health score were weak for SED and LPA but gradually strengthened with increasing time spent in moderate and vigorous-intensity PA (up to 4000–5000 CPM).
Aadland et al. [29]	CS	Norway, 841, 5th grade (10.2 ± 0.3 Y)	BMI, WC, BP, TC, TG, HDL-C, glucose, insulin, LDL-C, HOMA	The strongest associations with metabolic health were found for VPA, while weaker associations were found for MPA and LPA, and no associations were found for SED.
Bell et al. [30]	Cohort	UK, 1826, 12–15 Y	BMI, BP, TC, TG, HDL-C, LDL-C, glucose, insulin, CRP	The associations of PA with metabolic traits were small and more robust for higher MVPA than lower SED. The activity was most strongly associated with cholesterol content in VLDL-C and HDL lipoprotein particles, with TG content in all particle types.
Carson et al. [31]	CS	Canada, 787, 11 Y	BMI	No association was observed between SED and BMI z-score. Conversely, MVPA was consistently associated with BMI z-score.
Carson & Janssen [32]	CS	US, 2527, 6–19 Y	WC, SBP, non-HDL-C, CRP	No association was observed between overall volume and patterns of SED with cardiometabolic risk factors. Conversely, high television watching and low MVPA were independently associated with cardiometabolic risk factors.
Chinapaw et al. [33]	CS	Hungary/Netherlands, 142, 10–13 Y	BMI, WC, TC, TG, HDL-C, LDL-C, glucose, C-peptide	Although BMI and WC were higher in the most sedentary versus the least sedentary children, no further evidence was found to support that more sedentary children were at increased metabolic risk.
Colley et al. [34]	CS	Canada, 799, 6–19 Y	BMI, WC, BP, non-HDL-C	SED accumulated during the after-school period was associated with BMI and WC, independent of MVPA, in boys aged 11–14 years. No sedentary behavior variables were independently associated with any health marker in older or younger boys or girls of any age.
Colley et al. [35]	CS	Canada, 878, 6–11 Y	BMI, WC, BP, non-HDL-C	Directly measured MVPA and sleep were significantly associated with BMI, directly measured MVPA was significantly associated with WC.
Dalene et al. [36]	CS	Norway, 970 (6 Y)/ 2423 (9 Y)/1544 (15 Y)	BMI, WC	Substituting 10 min/day of SED with LPA was associated with higher WC in all age groups. Substituting 10 min/day of SED with MPA was associated with lower WC in 6- and 9-year-olds. Substituting 10 min/day of SED with VPA was associated with lower WC in 9- and 15-year-olds. Associations were similar with BMI as the outcome. In prospective analyses, substituting SED with LPA, MPA, or VPA at age 9 was not associated with BMI or WC at age 15.
del Pozo-Cruz et al. [37]	CS	New Zealand, 1812, 5–24 Y	BMI, sleep time	MVPA and SED were found to have a unique effect on BMI. Further, substituting SED with LPA or MVPA was associated with a favorable effect on BMI across all age groups, with MVPA having the strongest association.
Ekelund et al. [38]	CS	Denmark, 1092 (9–10 Y)/829 (15–16 Y)	BMI, WC, skinfold thickness (triceps, biceps, subscapular, suprailiac), BP, TG, HDL-C, glucose, insulin, CRF (ergometer cycle test)	PA and CRF were found to be separately and independently associated with individual and clustered metabolic risk factors in children.
Hansen et al. [39]	CS	10 countries, 10,836 (10–14.9 Y)/ 2393 (15–18.4 Y)	WC, BP, TG, HDL-C, LDL-C, glucose, insulin	Replacing SED and/or LPA with MVPA in children and adolescents was favorably associated with most markers of cardiometabolic risk. Efforts were aimed at replacing SED with active behaviors, particularly those of at least moderate intensity.
Katzmarzyk et al. [40]	CS	Australia/Brazil/Canada/ China/Colombia/Finland/ India/Kenya/Portugal/ South Africa/UK/US, 6539, 9–11 Y	BMI	Greater MVPA and VPA were both associated with lower odds of obesity independent of SED. SED was positively associated with obesity, but not independent of MVPA.
Kuzik et al. [41]	CS	Denmark/Estonia/Portugal/US, 4581, 5–18 Y	BMI, BP, TG, HDL-C, glucose, HOMA-IR	More MVPA was beneficial for metabolic health and weight status, whereas lower SED was beneficial for metabolic health alone, although associations were weak.
Loprinzi et al. [42]	CS	US, 2644, 6–17 Y	Height, weight, WC, %BF (by DEXA), skinfold thickness (triceps, subscapular), energy intake	The low proportion of children engaging in ≥ 60 min/day of MVPA and accumulating relatively more LPA than SED had the lowest DXA-BF%.
Mitchell et al. [43]	LS	US, 789, 9–15 Y	BMI	SED was associated with greater increases in BMI at the 90th, 75th, and 50th BMI percentiles between ages 9 and 15 years, independent of MVPA. No associations were observed between SED and changes at the 25th and 10th BMI percentiles.
Moore et al. [44]	LS	Brazil/Europe/ US, 11,588, 4–18 Y	WC, BP, TG, HDL-C, LDL-C, glucose, insulin	Substituting LPA with VPA was inversely associated with WC and insulin. However, VPA was inconsistently related to the remaining biomarkers after controlling for SED and MPA.
Moura et al. [45]	CS	Brazil, 84 (male), 14–18 Y	BMI, WC, BF%, BP, TC, TG, HDL-C, LDL-C, glucose, insulin, HOMA-IR, HOMA-*β*, HOMA2-S	Replacing SED with LPA showed positive results in HDL-C, HOMA2-S, and SBP, while replacing SED with MVPA was associated with only one obesity indicator (BF%).
Moura et al. [46]	CS	Brazil, 84 (male), 14–18 Y	BMI, WC, skinfold thickness (triceps), FM, TC, TG, HDL-C, LDL-C, non-HDL-C, HOMA-IR, HOMA-*β*, HOMA2-S	Sitting less and standing more may be an effective method to reduce cardiometabolic biomarker levels related to lipid metabolism (TC, TG, Non-HDL-C, LDL-C), regardless of MVPA.
Nguyen et al. [47]	CS	Vietnam, 617, high school students (13.9 ± 0.7 Y)	BMI, WC, BP, skinfold thickness (triceps, subscapular, abdominal, medial calf), TG, HDL-C, LDL-C	Elevated BP was the most common individual component of metabolic syndrome (21.5%), followed by hypertriglyceridemia (11.1%). The odds of metabolic syndrome among youth in the lowest PA group (<43 min of PA/day) were five times higher than those in the highest PA group (>103 min/day).
Rendo-Urteaga et al. [48]	CS	Austria/Belgium/France/ Germany/Greece/Hungary/Italy/Spain/Sweden, 769, 12.5–17.5 Y	BMI, WC, BP, skinfold thickness (biceps, triceps, subscapular, suprailiac), TC, TG, HDL-C, insulin, HOMA-IR, CRF (20 m shuttle run test)	A positive association was found between “PA ≥ 60 min/d; SED ≥ 2 h” and the ratio TC/HDL-c; a negative association was found between “MVPA ≥ 60 min/d; SED < 2 h” and ∑4Skinfolds. “SED ≥ 2 h/d” was associated with increased cardiometabolic risk, while “PA ≥ 60 min/d; SED < 2 h” had a protective effect against cardiometabolic risk. Adolescents should be encouraged to decrease SED and increase PA, especially VPA, to reduce cardiometabolic risk.
Saunders et al. [5]	CS	Canada, 522, 8–11 Y	BMI, WC, BP, TG, HDL-C, glucose, insulin, hs-CRP	Breaks in SED and the number of sedentary bouts lasting 1–4 min were associated with a reduced cardiometabolic risk score, lower BMI z-score in both sexes. The number of sedentary bouts lasting 5–9 min was negatively associated with WC in girls only, while those lasting 10–14 min was positively associated with fasting glucose in girls, with a BMI z-score in boys.
Stockwell et al. [49]	CS	UK, 118, 11–12 Y	BMI, WC, BF%, BP, TC, HDL-C, LDL-C, glucose	The number of breaks in sitting per day was significantly negatively associated with weight, BMI, WC, and %BF and significantly positively associated with TC and HDL. Total time spent in prolonged sitting bouts was significantly negatively associated with weight, BMI, WC, and %BF, and significantly positively associated with TC and HDL in both regression models.
Strizich et al. [50]	CS	US, 1426, 8–16 Y	BMI, WC, BP, TG, HDL-C, LDL-C, glucose, insulin, HbA1c, HOMA-IR, hs-CRP, inhibitor-1, E-selectin, sRAGE	Deleterious levels of HDL-C, TG, IR, CRP, and plasminogen activator inhibitor-1 were associated with lower levels of MVPA and higher levels of SED.
Treuth et al. [51]	CS	US, 130, 7–19 Y	BMI, FM, FFM, BF%	No associations between measures of body composition and time spent in an activity level were seen in boys. FM and BF% were positively correlated to SED for girls. In contrast, FM and BF% were negatively related to time spent in LPA for girls.
Verswijveren et al. [52]	CS	Australia, 169, 8–9 Y	TC, TG, HDL-C, LDL-C, IR, HOMA-IR	Replacing 10 min of SED with VPA was associated with lower TG in the whole sample. Replacing SED with VPA was associated with better HDL-C and TG in children with healthy weight. Replacing SED with MPA was associated with better HOMA-IR and HDL-C in children with a healthy weight and overweight, respectively. Substituting SED with VPA specifically accumulated in ≥1-min bouts was detrimentally associated with HOMA-IR in children with a healthy weight, but beneficially with the cardiometabolic summary score in the overweight sample.
White et al. [53]	CS	US, 3165, 6–18 Y	BMI, WC, WHR, BP, TC, TG, HDL-C, LDL-C, glucose, insulin	Longer continuous bouts of MVPA had lower BMI percentile, WC, WC percentile, and WHR than participating in shorter bouts of MVPA.

Abbreviations: BF, body fat; BMI, body mass index; BP, blood pressure; CPM, counts per minute; CRF, cardiorespiratory fitness; CRP, C-reactive protein; CS, cross-sectional study; DBP, diastolic blood pressure; FM, fat mass; FFM, fat free mass; HDL-C, high density lipoprotein cholesterol; hs-CRP, high sensitivity C-reactive protein; HOMA, homeostasis model assessment; HOMA-IR, homeostasis model assessment of insulin resistance; IR, insulin resistance; LDL-C, low density lipoprotein cholesterol; LPA, light physical activity; LS, longitudinal study; MPA, moderate physical activity; MVPA, moderate-to-vigorous physical activity; non-HDL-C, non-high density lipoprotein cholesterol; OW, overweight; PA, physical activity; SBP, systolic blood pressure; SED, sedentary time; TC, total cholesterol; TG, triglycerides; UK, United Kingdom; US, United States; VLDL-C, very low density lipoprotein cholesterol; VPA, vigorous physical activity; WC, waist circumference; WHR, waist to hip ratio; Y, years.

## Data Availability

The data presented in this study are available on request to the authors.

## Supplementary Materials

The following are available online at https://www.mdpi.com/article/10.3390/healthcare9060709/s1, Table S1: Methods used for examining the accelerometer data collection protocols and processing criteria.
