# Supplementary material for: Relationship of Physical Activity and Sedentary Time with Metabolic Health in Children and Adolescents Measured by Accelerometer: A Narrative Review
Source: Healthcare (Basel). 2021 Jun 10;9(6):709. doi: 10.3390/healthcare9060709 (PMC8230405; doi:10.3390/healthcare9060709)
Supplement: Supplementary file 1 [file healthcare-09-00709-s001.zip › healthcare-1213797-supplementary.pdf]

Table S1. Methods used for examining the accelerometer data collection protocols and processing criteria.

Reference	Outcome	Accelerometer	Cut-off point/definition of bouts and breaks	Epoch	Valid days/week	Valid h/day or min/day	Non-wear time
Aadland et al. [28]	Volume	ActiGraph (uniaxial)	SED: 0–99 CPM/LPA: 100–2295 CPM<br>MPA: 2296–4011 CPM/VPA: ≥4012 CPM	60s	≥4	600–1080 min	≥60 min
Aadland et al. [29]	Volume<br>PA_Bouts	ActiGraph (unknown)	- PA<br>SED: 0–99 CPM/LPA: 100–2295 CPM<br>MPA: 2296–4011 CPM/VPA: ≥4012 CPM<br>- PA_Bouts<br>• with ≥80% of bout duration<br>• 1 s, 2–4 s, 5–9 s, 10–19 s, 20–29 s, 30–39 s, 40–59 s, 1.0–1.4 min, 1.5–1.9 min, 2.0–2.9 min, 3–4 min, 5–9 min, 10–29 min, 30–59 min, ≥60 min	1s<br>10s<br>60s	≥4	≥480 min	≥60 min
Bell et al. [30]	Volume	ActiGraph (uniaxial)	SED: <199 CPM<br>MVPA: ≥3600 CPM	30 Hz	≥3	≥600 min	≥10 min
Carson et al. [31]	Volume<br>SED_Bouts<br>SED_Breaks	ActiGraph (unknown)	- PA<br>SED: ≤100 CPM<br>Brisk walking: 101 CPM to 4.0. METs<br>MVPA: ≥4.0 METs<br>- SED_Bouts: 1–4 min, 5–9 min, 10–19 min, 20–29 min, ≥30 min<br>- SED_Breaks: 5-s epoch change from SED to LPA or MVPA	5s	≥4	≥600 min	≥60 min
Carson & Janssen [32]	Volume<br>SED_Bouts<br>SED_Breaks	ActiGraph (uniaxial)	- PA<br>SED: 0–99 CPM/LPA: 100 CPM to 4.0 METs<br>MVPA: ≥4.0 METs<br>- SED_Bouts<br>≥30 min (with ≥80% of minutes below 100 CPM)<br>- SED_Breaks<br>• <80% of minutes below 100 CPM<br>• ≥5 consecutive min ≥100 CPM	60s	≥4	≥600 min	≥20 min
Chinapaw et al. [33]	Volume<br>SED_Bouts	ActiGraph (unknown)	- PA<br>SED: 0–99 CPM/MVPA: ≥3000 CPM<br>- SED_Bouts ≥10 min<br>(with 2 min tolerance)	15s	≥3 weekdays<br>+<br>≥8 h/day weekend day	≥600 min	≥20 min
Colley et al. [34]	Volume<br>SED_Bouts<br>SED_Breaks	Actical (unknown)	- PA<br>SED: ≤100 CPM/MVPA: ≥1500 CPM<br>- SED_Bouts<br>• Lasting a minimum of 1 min<br>(a transition from <100 to ≥100 CPM)<br>• ≥20, 40, 60, 80, 100, 120 min<br>- SED_Breaks<br>• <80% of minutes below 100 CPM<br>• ≥3 consecutive min ≥100 CPM<br>• ≥1500 CPM	60s	≥4	≥600 min	≥60 min
Colley et al. [35]	Volume	Actical (unknown)	SED: 0–99 CPM<br>MVPA: ≥1500 CPM	60s	≥4	≥600 min	≥60 min

Dalene et al. [36]	Volume<br>ISM_Bouts	ActiGraph (unknown)	- PA<br>SED: 0–99 CPM/LPA: 100–1999 CPM<br>MPA: 2000–5999 CPM/VPA: ≥6000 CPM<br>- ISM_Bouts 10 min	10s	≥2	≥600 min	≥20 min
del Pozo-Cruz et al. [37]	Volume<br>ISM_Bouts	ActiGraph (unknown)	- PA<br>SED: <1.5 METs (0–149 CPM)<br>LPA: 1.5≤ METs <3 (150–499 CPM)<br>MPA: 3≤ METs <6 (500–5999 CPM)<br>VPA: ≥6000 CPM<br>- ISM_Bouts<br>• 5–19 years age group: 60 min<br>• 20–24 years age group: 30 min	10s	≥3	≥600 min	≥20 min
Ekelund et al. [38]	Volume	ActiGraph (uniaxial)	SED: <500 CPM/LPA: 501–2000 CPM<br>MPA: 2001–3999 CPM/VPA: ≥4000 CPM	60s	≥3	≥600 min	≥10 min
Hansen et al. [39]	Volume<br>ISM_Bouts	ActiGraph (uniaxial)	- PA<br>SED: 0–25 counts/15s<br>LPA: 26–573 counts/15s<br>MPA: 574–1002 counts/15s<br>VPA: ≥1003 counts/15s<br>- ISM_Bouts: 10 min	60s	≥1	≥600 min<br><1080 min	≥60 min
Katzmarzyk et al. [40]	Volume	ActiGraph (unknown)	SED: <25 counts/15s<br>MVPA: ≥574 counts/15s<br>VPA: ≥1003 counts/15s	15s	≥4	≥600 min (at least 1 weekend day)	≥20 min
Kuzik et al. [41]	Volume	ActiGraph (unknown)	SED: 0–99 CPM/MPA: 3–5.9 METs<br>VPA: ≥6 METs/MVPA: ≥3 METs	60s	≥1	≥600 min	≥60 min
Loprinzi et al. [42]	Volume<br>ISM_Bouts	ActiGraph	- PA<br>SED: 0–99 CPM/LIPA: ≥100 CPM<br>MPA: ≥2220 CPM/VPA: ≥4136 CPM<br>- ISM_Bouts: 60 min	unknown	≥4	≥600 min	≥60 min
Mitchell et al. [43]	Volume	ActiGraph (unknown)	SED: 0–99 CPM/LPA: 100–2295 CPM<br>MVPA: ≥2296 CPM	60s	≥3	≥600 min	≥60 min
Moore et al. [44]	Volume<br>ISM_Bouts	ActiGraph (unknown)	- PA<br>• Pate's study: SED: 0–152 CPM<br>MPA: 1677–3364 CPM/VPA: ≥3365 CPM<br>• Evenson's study: SED: 0–100 CPM<br>MPA: 2296-4011 CPM/VPA: ≥4012 CPM<br>• ICAD's study: SED: 0–100 CPM<br>MPA: 3000–6000 CPM/VPA: ≥6001 CPM<br>- ISM_Bouts: 5, 10, 20, 50 min	60s	≥3	≥600 min	≥60 min
Moura et al. [45]	Volume<br>PA/SED_Bouts<br>ISM_Bouts	ActiGraph (triaxial)	- PA<br>SED: ≤720 CPM/LIPA: 721–3027 CPM<br>MVPA: ≥3028 CPM<br>- PA/SED_Bouts<br>≥10 min (drop time of 2 min)<br>- ISM_Bouts: 5, 10, 30, 60 min	15s	≥3	≥480 min	≥60 min
Moura et al. [46]	Volume (CPM, min/day)<br>Time blocks	ActiGraph (triaxial)	- PA<br>MVPA: ≥3028 CPM<br>- Sitting time<br>Sitting-High: ≥391.8 min/day	15s	≥3	≥480 min	≥60 min

			Sitting-Low: <391.8 min/day<br>- Standing time<br>Standing-High: ≥409.2 min/day<br>Standing-Low: <409.2 min/day<br>- Bouts: 15, 30, 60, 120 min				
Nguyen et al. [47]	Volume	ActiGraph (uniaxial)	Lowest activity: <43 min/day<br>MVPA: >103 min/day (≥3.0 MET)	60s	≥4	≥480 min	un-known
Rendo-Urteaga et al. [48]	Volume	- PA<br>ActiGraph (unknown)<br>+<br>Question-naire (IPAQ-A)<br>- SED<br>Question-naire (HELENA question-naire)	- PA<br>Inactive: 0–99 CPM<br>MPA: 2000–3999 CPM<br>VPA: ≥4000 CPM	15s	≥3	≥480 min	un-known
Saunders et al. [5]	Volume<br>SED_Bouts<br>SED_Breaks	ActiGraph (unknown)	- PA<br>SED: <100 CPM/LPA: 100–2296 CPM<br>MVPA: ≥2296 CPM<br>- SED_Bouts<br>• ≥1 min with 0–99 CPM<br>• Daily bouts of SED<br>: 1–4, 5–9, 10–14, 15–29, ≥30 min<br>- SED_Breaks<br>• Time lasting ≥1 min<br>• CPM rose to 100 or above	60s	≥4	≥600 min	≥60 min
Stockwell et al. [49]	Volume	activPAL	- Sitting_Bouts<br>≥30 min<br>- Sitting_Breaks<br>a non-sitting period in between two sit-ting bouts	15s	≥4 (include at least 1 weekend day)	≥600 min	un-known
Strizich et al. [50]	Volume	Actical	SED: <18 counts/15s<br>MVP: ≥441 counts/15s	unknown	≥3	≥480 min<br><1140 min	≥90 min
Treuth et al. [51]	Volume	Actiwatch (omnidirec-tional)	SED: <100 CPM/LPA: 100 to <900 CPM<br>MPA: 900 to <2200 CPM/VPA: ≥2200 CPM	60s	≥4 (with 2 weekend days)	≥1000 min	unknown
Verswijveren et al. [52]	Volume<br>ISM_Bouts	ActiGraph (uniaxial)	- PA<br>SED: ≤100 CPM<br>LPA: 1.5–3.99 METs<br>MPA: 4–5.99 METs<br>VPA: ≥6 METs<br>- ISM_Bouts<br>LPA, MPA, VPA bouts ≥1 min	15s	≥4	≥480 min (≥420 on weekday + weekend days)	≥20 min
White et al. [53]	Volume<br>PA_Bouts	ActiGraph (unknown)	- PA<br>1) Triano: SED: 0–99 CPM, LPA: 101–2019 CPM, MPA: 2020–5998 CPM, VPA: ≥5999 CPM<br>2) Freedson: SED: ≤149 CPM, LPA: 150–499 CPM, MPA: 500–3999 CPM, VPA: 4000–7599 CPM, VVPA: ≥7600 CPM	60s	≥1	≥600 min	≥60 min

	3) Evenson: SED: ≤25 counts/15s, LPA: 26–573 counts/15s, MPA: 574–1002 counts/15s, VPA: ≥1003 counts/15s<br>- PA_Bout<br>Sporadic: 1–4.9 min of continuous MVPA<br>Short: 5–9.9 min of continuous MVPA<br>Medium-long: ≥10 min of continuous MVPA	

Abbreviations: CPM, counts per minute—an indicator of the total volume of physical activity (e.g., average intensity of PA); HELENA, healthy lifestyle in Europe by nutrition in adolescents; IPAQ-A, international physical activity questionnaire for adolescents; ISM, isotemporal substitution model; LPA, light-intensity physical activity; METs, metabolic equivalents; MPA, moderate-intensity physical activity; MVPA, moderate-to-vigorous physical activity; PA, physical activity; SED, sedentary time; SVMgs, signal magnitude vector (gravity-subtracted) expressed in 1s epochs; VPA, vigorous physical activity; VVPA, very vigorous physical activity
